# Toward Prediction of Financial Crashes with a D-Wave Quantum Annealer

**DOI:** 10.3390/e25020323

**Published:** 2023-02-10

**Authors:** Yongcheng Ding, Javier Gonzalez-Conde, Lucas Lamata, José D. Martín-Guerrero, Enrique Lizaso, Samuel Mugel, Xi Chen, Román Orús, Enrique Solano, Mikel Sanz

**Affiliations:** 1 International Center of Quantum Artificial Intelligence for Science and Technology (QuArtist) and Department of Physics, Shanghai University, Shanghai 200444, China; 2 Department of Physical Chemistry, University of the Basque Country UPV/EHU, Apartado 644, 48080 Bilbao, Spain; 3 ProQuam Co., Ltd., Shanghai 200444, China; 4 Quantum Mads, Uribitarte Kalea 6, 48001 Bilbao, Spain; 5 EHU Quantum Center, University of the Basque Country UPV/EHU, Apartado 644, 48080 Bilbao, Spain; 6 Departamento de Física Atómica, Molecular y Nuclear, Universidad de Sevilla, 41080 Sevilla, Spain; 7 Instituto Carlos I de Física Teórica y Computacional, 18071 Granada, Spain; 8 IDAL, Electronic Engineering Department, University of Valencia, Avgda. Universitat s/n, 46100 Burjassot, Spain; 9 ValgrAI: Valencian Graduated School and Research Network of Artificial Intelligence, Camí de Vera, s/n, Edificio 3Q, 46022 Valencia, Spain; 10 Multiverse Computing, Pio Baroja 37, 20008 San Sebastián, Spain; 11 Donostia International Physics Center, Paseo Manuel de Lardizabal 4, 20018 San Sebastián, Spain; 12 IKERBASQUE, Basque Foundation for Science, Plaza Euskadi 5, 48009 Bilbao, Spain; 13 Kipu Quantum, Greifswalderstrasse 226, 10405 Berlin, Germany; 14 Basque Center for Applied Mathematics (BCAM), Alameda de Mazarredo 14, 48009 Bilbao, Spain

**Keywords:** quantum computation, financial networks, adiabatic quantum optimization

## Abstract

The prediction of financial crashes in a complex financial network is known to be an NP-hard problem, which means that no known algorithm can efficiently find optimal solutions. We experimentally explore a novel approach to this problem by using a D-Wave quantum annealer, benchmarking its performance for attaining a financial equilibrium. To be specific, the equilibrium condition of a nonlinear financial model is embedded into a higher-order unconstrained binary optimization (HUBO) problem, which is then transformed into a spin-1/2 Hamiltonian with at most, two-qubit interactions. The problem is thus equivalent to finding the ground state of an interacting spin Hamiltonian, which can be approximated with a quantum annealer. The size of the simulation is mainly constrained by the necessity of a large number of physical qubits representing a logical qubit with the correct connectivity. Our experiment paves the way for the codification of this quantitative macroeconomics problem in quantum annealers.

## 1. Introduction

Economics is a complex science in which the agents’ psychology plays an essential role that is often hardly grasped by mathematical models. However, economists persist in trying to predict market behavior employing sophisticated models, which has resulted in the field of quantitative finance. Following this idea, quantitative finance and economics emerged, which were applied to understand the evolution of financial markets and economies, as well as provide forecasts. A realistic question in risk management is whether there would be a drastic drop in market values if the prices of assets suffered some small perturbations. The cross-holdings and nonlinear nature of financial network dynamics can cause chain reactions, implying that sudden drops in market value might affect other nodes in the network resulting in a financial crisis. Presently, the prediction of crashes is mainly performed by studying previous cases in history and comparing these with the current configuration [1,2,3,4,5,6]. Although this empirical approach has been successful [7], the economic environment is constantly evolving. Hence, we cannot limit ourselves to predicting economic disasters that are qualitatively similar to past events. Therefore, ab initio simulations of financial networks will become essential for avoiding financial crises. This problem was recently shown to be NP-hard [8]. Therefore, given the current standpoint on complexity theory, this problem is not expected to be efficiently solvable by a classical computer. Indeed, given the global knowledge of a financial network, the time to compute the consequences of a perturbation would far exceed the age of the universe.

An alternative approach to this problem was presented in Refs. [9,10], where ways to tackle this type of problem using quantum annealers were presented. In particular, a mathematically identical problem was simulated, and the corresponding results measured [11,12,13,14]. Specifically, it was shown that obtaining the equilibrium configuration of a financial network is equivalent to solving a higher-order unconstrained binary optimization (HUBO) problem, which should be feasible for a quantum annealer that allows for multi-qubit interactions. Unfortunately, this hardware has not been realized yet, as state-of-the-art quantum annealers are restricted to two-qubit interactions [15]. A possible workaround, which comes at the cost of introducing ancillary qubits, is to find an effective Hamiltonian with the same low-energy subspace and two-qubit interactions at most. This leaves us with the problem of solving a quadratic unconstrained binary optimization (QUBO) problem, whose optimum encodes the equilibrium configuration of a financial network. This problem can be addressed by employing a quantum annealer. The D-Wave 2000Q quantum annealer equipped with a Chimera architecture requires a large number of physical qubits to obtain the desired connectivity and limits the number of institutions and assets considered. An analysis of the changes experienced by the financial network to reach its equilibrium configuration will determine whether a crash has occurred.

In this paper, we experimentally implement the study presented in Refs. [9,10]. Specifically, we compute the equilibrium configuration of a financial network before and after a perturbation with a D-Wave 2000Q quantum annealer and compare the results to alternative methods. Although the D-Wave machine has been successfully used to solve problems in engineering [16], cryptography [17], biology [18], and quantitative finance [19,20], among others, it is the first time that quantum annealing is applied to solve a macroeconomic problem. This should attract more attention from the finance and economic disciplines towards the potential of quantum computing [21,22,23,24,25,26,27].

The contents are organized as follows. In Section 2, we introduce the model of the financial network that will be considered. Section 3 reviews the quantum annealing algorithm to find financial equilibrium. Section 4 experimentally proves the validity of the scheme by finding the financial equilibrium of a random network of the largest implementable size using a D-Wave 2000Q quantum annealer; for this network, we also experimentally show how the scheme allows for the computation of the financial equilibrium. Section 5 analyzes the achieved results and discusses possible further improvements. The conclusions drawn from the work are presented in Section 6.

## 2. Formulation of the Model

**Figure 1:**
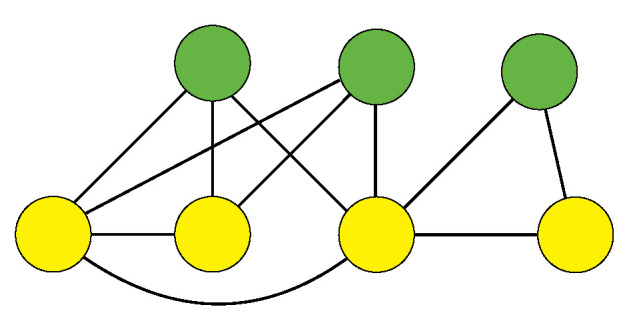
Example of a financial network: the yellow and green nodes denote institutions and assets, respectively. The links denote the ownerships and cross-holdings described by the ownership matrices D and C, respectively. The diagonal matrix $\tilde{C}$ represents the self-ownership of institutions, which would be plotted as self-loops in the graph representation. The equity value $V_{i}$ of institution *i* is defined by summing its ownership of all assets and cross-holdings.

A nonlinear network model for financial markets is proposed in Ref. [9]. It is made up of *n* institutions and *m* assets and aims to represent the market values of institutions by mapping them onto a graph, as shown in Figure 1. We codify the prices of the *m* assets by an m−dimensional vector $\vec{p}\in R^{m}$, where the element $p_{k}$ represents the price of asset *k*. Moreover, an n×m ownership matrix D can be defined such that the element $D_{ik}\geq 0$ corresponds to the percentage of asset *k* owned by institution *i*. There is also an n×n ownership matrix C that describes the cross-holdings and self-ownerships among institutions. The coefficients $C_{ij}$ denote the percentage of institution *j* owned by institution *i*. By considering all self-ownerships (i.e., the diagonal elements) from C, one forms a new diagonal matrix $\tilde{C}$, which represents the self-ownership only such that the matrix $C=C-\tilde{C}$ codifies all cross-holdings. The equity value $V_{i}$ of institution *i* is defined by summing its ownership of all assets and cross-holdings, $V_{i}=\Sigma_{k}D_{ik}p_{k}+\Sigma_{j}C_{ij}V_{j}$. One thus obtains a matrix equation $\vec{V}=D\vec{p}+C\vec{V}$, where the equity value vector $\vec{V}\in R^{n}$ is an n−dimensional vector. Accordingly, the market value is the equity value rescaled with its self-ownership, resulting in the n−dimensional market value vector $\vec{v}=\tilde{C}\vec{V}$. The solution to the linear matrix equation thus reads

(1) $$\vec{v}=\tilde{C}{\left(I-C\right)}^{-1}D\vec{p}.$$

We introduce the nonlinear effect of *panic* in the model via a Heaviside-theta function Θ; if the market value $v_{i}$ drops below the critical value $v_{c}^{i}$, *failure* of institution *i* occurs and its equity value drops by $\beta_{i}\left(\vec{p}\right)$, which is governed by the price vector of assets. Once we define the failure vector $\vec{b}\left(\vec{v},\vec{p}\right)=\vec{\beta }\left(\vec{p}\right)\circ \left(1-\Theta \left(\vec{v}-\vec{v_{c}}\right)\right)$, where ∘ denotes the Hadamard product, the market value vector with nonlinearity can be written as

(2) $$\vec{v}=\tilde{C}{\left(I-C\right)}^{-1}\left(D\vec{p}-\vec{b}\left(\vec{v},\vec{p}\right)\right).$$

Mathematically, it is the nonlinearity of $\vec{b}\left(\vec{v},\vec{p}\right)$ that makes financial networks so hard to predict. This drop may cause an institution’s value to *crash*, a behavior that can infect other nodes in the network. Under our definition, a financial crash happens when the market value of an institution, considering the nonlinear term, is lower than the pre-perturbation prices calculated using the linear model as a result of a perturbation in the asset prices.

## 3. Quantum Annealing Algorithm

As proposed in Ref. [9], finding financial equilibrium can be presented as the minimization of an objective function, which is equivalent to finding the ground state of a classical spin Hamiltonian.

By squaring Equation (2), we obtain an objective function that meets its minimum value when the market value state is set to be the equilibrium state

(3) $$\mathrm{Obj}\left(\vec{v}\right)={\left(\vec{v}-\tilde{C}{\left(I-C\right)}^{-1}\left(D\vec{p}-\vec{b}\left(\vec{v},\vec{p}\right)\right)\right)}^{2}.$$

Thus, our task is now to find the $\vec{v}$ that minimizes $\mathrm{Obj}(\vec{v})$ for a given financial network, which is an NP-hard problem [28].

Next, we need to deal with the nonlinear terms (modeling failure) of the objective function so that once the objective function is transformed to a spin-1/2 Hamiltonian, it should ideally be made of polynomial terms only due to the limitations of quantum annealers. Thus, one expands the failure terms with Heaviside-theta functions in terms of polynomials. This expansion is not unique and here, we choose the Legendre expansion [9],

(4) $$\Theta \left(x\right)=\frac{1}{2}+\sum_{l=1}^{\infty }\left(P_{l-1}\left(0\right)+P_{l+1}\left(0\right)\right)P_{l}\left(x\right),$$

in the domain [−1,1], where $P_{l}\left(x\right)$ is the *l*-th Legendre polynomial. By setting $x=\left(v_{i}-v_{i}^{c}\right)/v_{max}^{i}$, Equation (4) enables us to expand $\Theta (v_{i}-v_{i}^{c})$ in the range of $v_{i}\in \left[0,v_{max}^{i}\right]$. Using this expansion as an example, we take the approximation

(5) bi(vi,p→)≈βi(p→)12−∑l=0rΓl2l∑k=0llkl+k−12lv¯ik

where $\Gamma_{l}=\frac{\sqrt{\pi }}{2\Gamma \left(\frac{2-l}{2}\right)\Gamma \left(\frac{3+l}{2}\right)}$ and ${\bar{v}}_{i}=\frac{v_{i}-v_{i}^{c}}{v_{\max}^{i}}$. The polynomial expansion removes the discontinuity while maintaining the strong nonlinearity of the network.

We now encode the continuous variables $v_{i}$ with classical bits. This will allow us to rewrite the resulting objective function in digital form. The expansion is straightforward and reads $v_{i}=\sum_{\alpha =-\infty }^{\infty }x_{i,\alpha }2^{\alpha }$. However, due to the limited resources in real-world devices, one must truncate this expansion, i.e., $v_{i}\approx \sum_{\alpha =-q}^{q}x_{i,\alpha }2^{\alpha }$, where $x_{i,\alpha }$ are classical bits with binary values of 0 or 1. In this way, the market value of institution *i* is encoded with 2q+1 classical bits. The maximal market value $v_{i}^{max}$ is given by $\sum_{\alpha =-q}^{q}2^{\alpha }$.

Considering (vi−vic)k=∑h=0k(−1)hkhvik−h(vic)h and vin=∑m0+...+mp=nnm0,...,mp$\prod_{0\leq \alpha \leq p}^{m_{\alpha }\neq 0}2^{\alpha m_{\alpha }}x_{i,\alpha }$, the resulting objective function is a polynomial of the binary variables $x_{i,\alpha }$ of degree 2r.

(6) $$\hat{H}=\sum_{i}{\left(\sum_{\alpha =-q}^{q}x_{i,\alpha }2^{\alpha }-\gamma_{i}+\sum_{j}{\bar{C}}_{ij}b_{j}\left(x_{j,\alpha },\vec{p}\right)\right)}^{2}$$

with $\gamma_{i}=\sum_{j}{\bar{C}}_{ij}\sum_{k}D_{jk}p_{k}$ and ${\bar{C}}_{ij}=\tilde{C_{ii}}{\left(I-C\right)}_{ij}^{-1}$. To express this as a spin-1/2 Hamiltonian, we replace the binary variables $x_{i,\alpha }$ with qubit operators ${\hat{x}}_{i,\alpha }$ with eigenvalues of 0 and 1, i.e., ${\hat{x}}_{i,\alpha }|0\rangle =0$, ${\hat{x}}_{i,\alpha }|1\rangle =|1\rangle$. The Pauli-*z* operator satisfies ${\hat{x}}_{i,\alpha }=\left(1+{\hat{\sigma }}_{i,\alpha }^{z}\right)/2$ and, therefore, the Hamiltonian encodes the objective function but is written with Pauli matrices, including all types of multi-spin interactions up to 2r-body terms.

**Figure 2:**
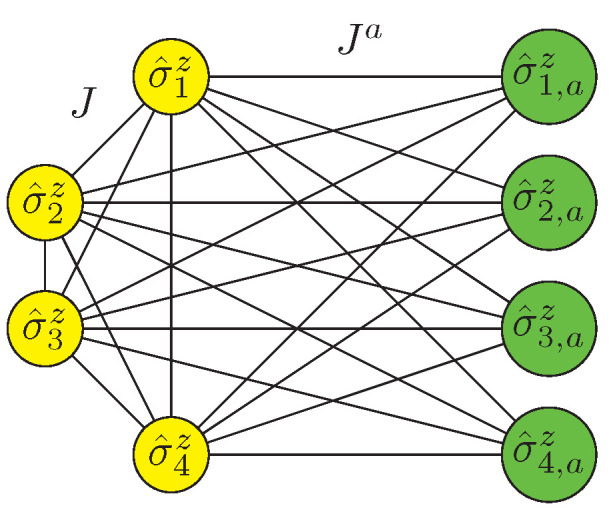
Recast of quantum Hamiltonian with *k*-qubit interactions into a modified, effective Hamiltonian with the same low-energy spectrum with two-qubit interactions at most. We illustrate the particular case of a k=4-qubit interaction, which requires the introduction of 4 ancilla qubits to obtain the effective Hamiltonian.

The Hamiltonian obtained is appropriate for a quantum annealer that allows many-qubit interactions. However, state-of-the-art quantum annealers only accept inputs with, at most, two-qubit interactions. Finding the ground state of a spin-1/2 Hamiltonian is equivalent to solving a quadratic unconstrained binary optimization (QUBO) problem, which is the input of the quantum annealer. Thus, we should recast our quantum Hamiltonian into a modified, effective Hamiltonian with, at most, two-qubit interactions. Some protocols achieving exactly this are proposed in Refs. [29,30,31,32,33,34,35]. In particular, we base our protocol on Ref. [35], where *k* ancilla qubits are introduced to implement an effective *k*-qubit interaction. Suppose that there is a *k*-qubit interaction term ${\hat{H}}_{k}=J_{k}\Pi_{i=1}^{k}\sigma_{i}^{z}$ with the same low-energy spectrum of another Hamiltonian term ${\tilde{H}}_{k}$ with, at most, two-qubit interactions. We can express ${\tilde{H}}_{k}$ with *k* logical qubits and *k* extra ancilla qubits as

(7) $$\begin{array}{ccc} & & {\tilde{H}}_{k}=J\sum_{i=2}^{k}\sum_{j=1}^{i-1}{\hat{\sigma }}_{i}^{z}{\hat{\sigma }}_{j}^{z}+h\sum_{i=1}^{k}{\hat{\sigma }}_{i}^{z}\\ & & +J^{a}\sum_{i=1}^{k}\sum_{j=1}^{k}{\hat{\sigma }}_{i}^{z}{\hat{\sigma }}_{j,a}^{z}+\sum_{i=1}^{k}h_{i}^{a}{\hat{\sigma }}_{i,a}^{z}, \end{array}$$

as represented in Figure 2. This two-qubit Hamiltonian has the same low-energy spectrum as ${\hat{H}}_{k}$ when *J*, $J^{a}$, *h*, and $h_{i}^{a}$ are set to appropriate values. As Ref. [35] suggested, this can be achieved once $q_{i}={\left(-1\right)}^{k-i+1}J_{k}+q_{0}$, $h=-J^{a}+q_{0}$, $h_{i}^{a}=-J^{a}\left(2i-k\right)+q_{i}$ and $J=J^{a}$, with any $q_{0}$ that satisfies $|J_{k}|\ll q_{0}<J^{a}$ and $|J_{k}|\ll J^{a}-q_{0}<J^{a}$. These conditions can be relaxed to $|J_{k}|<q_{0}<J^{a}$ and $|J_{k}|<J^{a}-q_{0}<J^{a}$ if one aims to have only the same ground state rather than the whole low-energy sector. We depict the low-energy spectrum of this two-qubit Hamiltonian for k=3 logical qubits in Table 1.

## 4. Implementation in a D-Wave 2000Q Quantum Annealer

**Figure 3:**
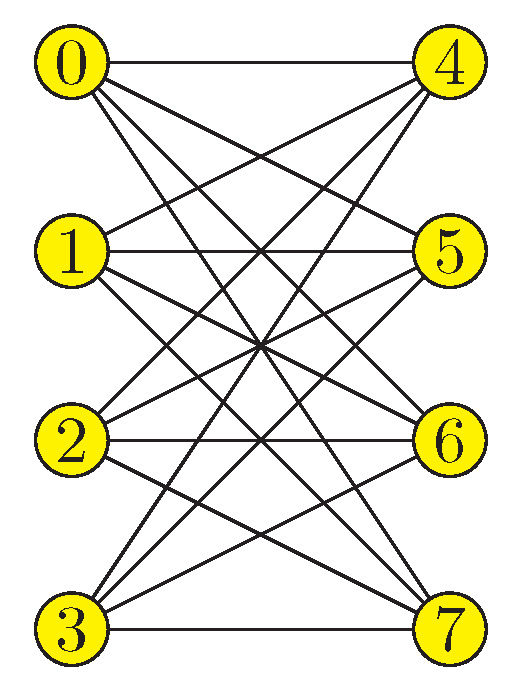
Chimera graph topology produced by the D-Wave 2000Q quantum annealer. The 2048 qubits are partitioned into subgraphs of 8 qubits. The connection between subgraphs is sparse; in each of these subgraphs there are two sets of four qubits and each qubit connects to all qubits in the other set but to none in its own, forming a $K_{4,4}$ bipartite graph.

Once shown that it is possible to recast the problem of finding financial equilibrium into a language amenable to QUBO solvers and, in particular, quantum annealers, this section deals with its implementation using a state-of-the-art quantum annealer, namely the D-Wave 2000Q. This quantum annealer consists of up to 2048 qubits connected according to the Chimera graph topology (see Figure 3). It is designed to solve embedded Ising problems or QUBO problems.

Two simulations were produced:

1. A financial network without a failure term, which is simple to solve on a classical computer in order to benchmark the performance of the quantum processor.
2. A financial network with an inherently nonlinear risk of failure. We perturb the asset price vector in this network to compute the new equilibrium configuration using the quantum annealing algorithm.

**Figure 4:**
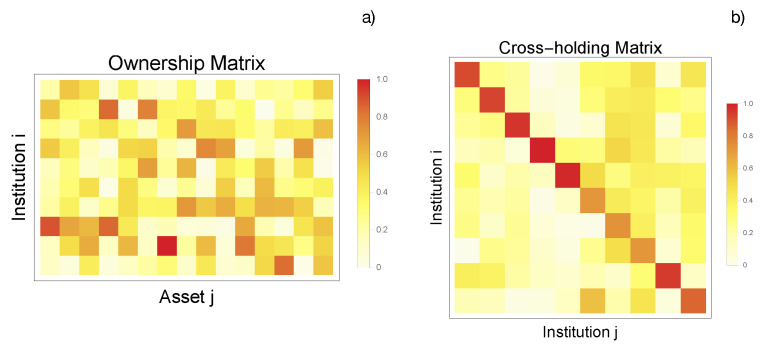
(**a**) Ownership matrix D for the linear model. The element $D_{ik}\geq 0$ corresponds to the percentage of asset *k* owned by institution *i*. We randomize the ownership matrix D with a Dirichlet distribution that satisfies $\sum_{i=1}^{n}D_{ij}=1$. (**b**) Cross-holding matrix C for the linear model that describes the cross-holdings and self-ownerships among institutions. The cross-holding matrix is generated in a similar way to the ownership matrix but with a constraint that all diagonal elements should be larger than 0.5, ensuring that all institutions can make decisions according to their own wills. These data, as well as the asset prices, have been synthetically produced but following all constraint conditions proposed in the theoretical model [9].

We initially generate a financial network with 10 institutions and 15 assets. To demonstrate the algorithm, we randomize the ownership matrix D with a Dirichlet distribution that satisfies $\sum_{i=1}^{n}D_{ij}=1$, where $D_{ij}$ are random variables. The cross-holding matrix C is generated in a similar way but with the constraint that all diagonal elements should be larger than 0.5, ensuring that all institutions can make decisions according to their own wills. Thus, we randomize ${\tilde{C}}_{ii}$ between 0.5 and 1 and randomize $\sum_{i=1}^{n}C_{ij}=1-{\tilde{C}}_{jj}$ with a rescaled Dirichlet distribution. The price vector $\vec{p}$ is also random, with $p_{i}\in \left[10,40\right]$. The network configuration is shown in Figure 4a,b.

We can calculate the equilibrium state $\vec{v_{q}}$ and the equity value vector $\vec{V}$ on a classical computer using

(8) $$\begin{array}{c} \vec{v_{q}}=\tilde{C}{\left(I-C\right)}^{-1}D\vec{p}, \end{array}$$

(9) $$\begin{array}{c} \vec{V}={\left(I-C\right)}^{-1}D\vec{p}. \end{array}$$

which are linear equations that, in fact, can be implemented in a quantum annealer using only 2-local terms, as a result of squaring the expression in a similar way to Equation (3).

The objective function shown in Equation (3) was implemented, for benchmarking reasons, both in a quantum annealer and a classical simulator. The variables $v_{i}$ were encoded as $v_{i}=\sum_{\alpha =0}^{6}2^{\alpha }x_{i,\alpha }$ on seven qubits. As such, this constrains the $v_{i}$ to be integers smaller than 127. A quantum implementation of this algorithm does not require ancilla qubits, as there are no many-qubit interactions.

**Figure 5:**
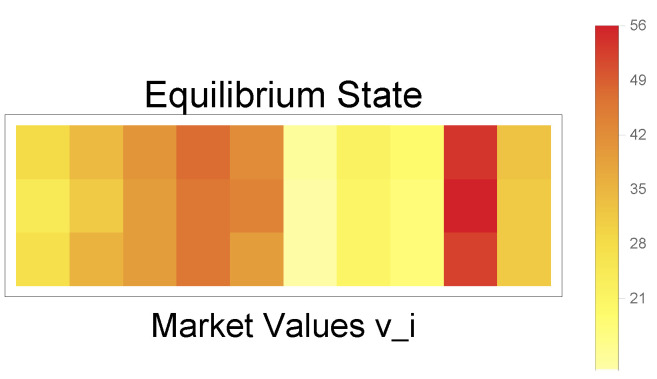
Linear model results. The first row shows the results when the matrix equation is solved exactly, the second row when *qbsolv* with the tabu classical solver is used, and the third row when *qbsolv* with the D-Wave 2000Q solver is employed. By comparing the individual equilibrium values, we can see that the quantum annealer provides a compatible solution to the exact solution.

The QUBO for this linear problem is a 70×70 matrix, with 210 couplers, which cannot be solved directly due to the topology structure of the quantum annealer. D-Wave provides software named *qbsolv*, which allows its quantum annealer to be used with a classical computer by splitting the QUBO matrix into partition matrices that can be embedded in the quantum annealer. As a decomposing solver, it finds a minimum value of a large QUBO problem by splitting it into pieces and solving it either via a D-Wave system or a classical tabu solver (both approaches were considered here for comparison purposes). Since the D-Wave 2000Q processor is a quantum annealer, 20 results would be obtained from a *qbsolv* process with a default setting; these results should be handled by a correction process, e.g., majority voting, to help us identify the most plausible answer. The result of this QUBO problem is shown in Figure 5, where the exact solution of a linear matrix equation, *qbsolv* solution using a classical tabu solver and *qbsolv* solution with the D-Wave quantum annealer are compared. By comparing the individual equilibrium values, it can be observed that the quantum annealer provides a solution that presents more accurate individual values of the assets than the prediction using the classical solver.

Although the failure-free model only has linear and quadratic terms in $v_{i}$, the nonlinear model has powers of $v_{i}$ up to order 2r. For large *r*, this can be extremely resource-consuming in terms of ancillary qubits due to the requested connectivity. An estimation of the number of qubits can be made by counting the number of interaction terms. Our Hamiltonian $\hat{H}$ can have up to ∑α=02rn(2q+1)α terms, where n(2q+1) denotes the logical qubits that are required. In each term, 3-to-2r new ancilla qubits are needed depending on the number of logical qubits in this term. Therefore, the number of necessary qubits grows rapidly with the degree of the polynomial expansion *r*. Note that the aforementioned QUBO problem is NP-hard for any n≥2. In practice, this is an upper bound of the required resources, calculated assuming that $\hat{H}$ has all possible terms up to order O(2r).

**Figure 6:**
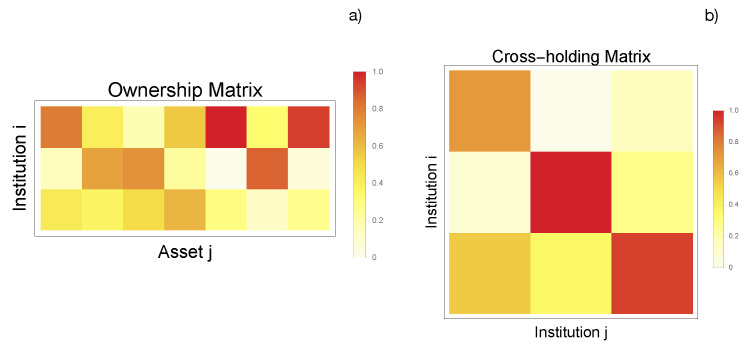
(**a**) Ownership matrix D for the implemented network with failure terms. The element $D_{ik}\geq 0$ corresponds to the percentage of asset *k* owned by institution *i*. We randomize the ownership matrix D with a Dirichlet distribution that satisfies $\sum_{i=1}^{n}D_{ij}=1$. (**b**) Cross-holding matrix C for the implemented network with failure terms describing the cross-holdings and self-ownerships among institutions. The cross-holding matrix is generated in a similar way to the ownership matrix but with a constraint that all diagonal elements should be larger than 0.5, ensuring that all institutions can make decisions according to their own wills.

Here, we implement an enhanced model with failure terms on the basis of the linear model previously simulated. We perturb the vector of asset prices, leaving the ownership matrix D and cross-holding matrix C invariant, and recompute the equilibrium state. Specifically, we set the price of some random assets to zero (to simulate, e.g., the assets’ destruction). In this study, we use an expansion of $\hat{H}$ to third order, which still characterizes the phenomenon of a sudden drop near the critical value. Moreover, this approach provides strong nonlinearity while saving plenty of qubit resources. As a result, 70 logical qubits and 872,690 ancilla qubits are required, which leads to a QUBO matrix of 872,760 × 872,760 entries, although only a minority of them, 4,446,575 couplers, are non-zero. Storing this sparse matrix results in the requirement of about 6TB RAM since each element has an accuracy of double float in *qbsolv*. Due to the limitations of state-of-the-art techniques, the network is reduced to three institutions, and each market value $v_{i}$ is encoded by five qubits, bounding the maximum market value to be 31. A new 3×7 ownership matrix D and a 3×3 cross-holding matrix C are generated and the price vector $\vec{p}$ before perturbation is $\vec{p}={\left\{8.43,\;14.47,\;6.75,\;8.09,\;19.11,\;11.32,\;7.19\right\}}^{T}$. The network configuration is shown in Figure 6a,b. The equilibrium state before perturbation without nonlinearity is given as $\vec{v_{q}}={\left\{21.18,23.33,\;30.83\right\}}^{T}$ and the critical value vector is set at 80% of the original equilibrium state, whereas the failure strength $\vec{\beta }$ is considered to be 30% of the original equity value. The corresponding perturbed price vector is given as $\vec{p}={\left\{8.43,\;14.47,\;0,\;8.09,\;0,\;11.32,\;7.19\right\}}^{T}$. Before calculating the new equilibrium state with the nonlinearity and perturbation, some parameters such as $J^{a}$ and $q_{0}$ must be set. The minor embedding of a submatrix in the D-Wave quantum annealer is performed by introducing a penalty function between qubits in the Chimera graph requiring $J^{m}\geq J^{a}$, which means that the $J^{a}$ for mapping multi-qubit interactions to two-qubit interactions should be on the proper scale. Meanwhile, as we mentioned earlier, we need to sample out the thermal fluctuation by assuming that $|{\hat{H}}_{k}|$ is much smaller than $J^{a}$ or the protocol will break down because those ancilla qubits will no longer be in the corresponding ground state. Thus, in the implementation, we took $J^{a}=20J_{k}$ and $q_{0}=10J_{k}$ to ensure that either $q_{0}$ or $J^{a}-q_{0}$ was at least 10 times larger than $J_{k}$.

**Figure 7:**
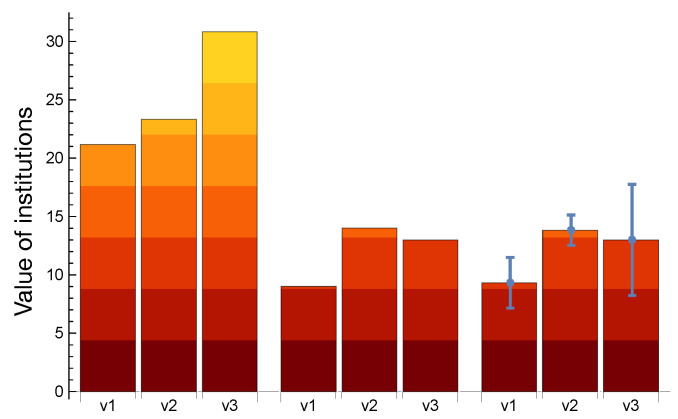
Market values $v_{1}$, $v_{2}$, and $v_{3}$ of institutions 1, 2, and 3, respectively, for different scenarios. The first group (**left**) is the equilibrium state without taking nonlinearity terms (perturbations) into consideration, where the asset price is calculated by inverting the matrix of Equation (1). The second group (**center**) is the equilibrium state after taking nonlinearity as the ‘failure term’, which is activated by a critical value vector of 80% of the original equilibrium state calculated with a straightforward method by trying $32^{3}$ times by brute force, corresponding to all possible combinations. The third group (**right**) shows the outcome of the *qbsolv* software in D-Wave 2000Q. The error bar characterizes a 95% confidence interval. The agreement between the integer and annealer solutions confirms the feasibility and accuracy of the method. Additionally, by comparing both solutions with the pre-perturbation values, we can conclude that we have detected the financial crash.

For this problem, the QUBO matrix had a size of 8280×8280, with 15 logical qubits, 8265 ancilla qubits, and 38,790 couplers. Note that the available quantum annealer structure is not optimized for this problem and the translation is not efficient because of the sparse connectivity of the quantum processor. Finally, we compare our results from the quantum annealer with the integer equilibrium solution calculated using the straightforward method by trying $32^{3}$ times, as shown in Figure 7, which shows the good agreement and accuracy of the proposed method. Comparing the results after the perturbation with the pre-perturbation values, we can conclude that we have detected the financial crash.

## 5. Results and Discussion

D-Wave is a quantum annealer designed to deal with an Ising model and QUBO problems. However, the problem faced in this paper, namely financial crisis prediction with nonlinearity associated with panic, is not QUBO but rather HUBO, thus requiring multi-qubit interactions. In order to approximate this HUBO problem with two-qubit interactions, with the current state of hardware and software, we were limited to simulating a small financial network made up of three institutions and cross-holdings.

An effective two-qubit quantum Hamiltonian could still not be read directly by the D-Wave system, which requires a QUBO- or Ising-type input. Although this can be generated by some open-source software such as *pyqubo*, the input size must be very small in order to avoid a stack overflow associated with recursion errors. A possible solution is to produce a Mathematica script that reads each term and writes it as a string of coefficients and qubits in an input file for the D-Wave system. Once we generate the input for this problem, it is still too large to be embedded in the D-Wave 2000Q quantum annealer because of the graph structure. Thus, *qbsolv* is an inevitable option for us, which works by separating the large matrix into submatrices and solving them using a classical tabu solver or D-Wave solver. This kind of hybrid computation provides the possibility to solve the complicated problem but brings some new constraints, namely (i) *Local hardware.* Once the QUBO matrix is provided, *qbsolv* allocates dynamic memory before separating it into submatrices with elements of double-precision floats by requiring a size of 8n×n bytes of memory. However, the bottleneck is not the memory size but the performance of the CPU since a large QUBO matrix will consume exhaustive CPU time if one needs high accuracy of the optimized results; (ii) *Algorithm*. Instead of a real quantum annealing process for the whole matrix, *qbsolv* provides a tabu algorithm or the D-Wave 2000Q quantum annealer for submatrices. The partition strategy for generating submatrices may get stuck in a local minimum instead of the global minimum that quantum annealing guarantees with high probability under ideal conditions, i.e., in the absence of decoherence and the adiabatic limit. Considering that the logical qubits only encode less than 1% in the QUBO matrix, the risk of getting stuck is still high, even if we sample over the thermal distribution or give a huge repeat limitation in the main loop to improve its accuracy. We would have to customize a random seed for the separation and check the final results manually to see whether they are near the equilibrium. Another option is to send the QUBO matrix to the solver many times and average the results to obtain the best solution; and (iii) *Quantum annealer.* The submatrices will be sent to the D-Wave 2000Q quantum annealing device for optimization after they are generated by Glover’s algorithm [36]. In the quantum annealing process, magnetic fields are applied to the processors and the strength should be accurate because $J^{k},J^{a}$ in the QUBO matrix and $J^{m}$ for the embedding belong to different magnitudes. Any imprecision in the system preparation will cause significant deviations from the correct results.

In this implementation, the accuracy is not especially high since we are not optimizing the objective function rigorously because the market values are the integers $v_{i}\in \left[0,v_{max}\right]$, which are constrained for the qubits we take to encode them. The computation time is also long, considering that there is a straightforward but equivalent classical algorithm by testing the value of the objective function $32^{3}$ times by brute force, corresponding to all possible combinations. Although mapping it to a QUBO problem and optimizing it with a general quantum annealer is not efficient enough for current technology, we believe it is a valuable example of how one can solve an NP-hard problem via quantum computation. With quantum annealers designed for solving HUBO problems that allow the implementation of multi-qubit interactions, we would avoid the overhead of resources and may obtain a speed-up factor in forecasting the behavior of complex financial networks over the use of general-purpose annealers. We expect this kind of quantum solver may be available in the near future. Meanwhile, D-Wave has recently announced its next generation of quantum annealers called the Advantage system [37], which consists of more than 5000 qubits connected with each other according to the Pegasus topology. In this manner, one could improve the number of qubits and the connectivity by a factor of 2.5.

Considering that a specialized quantum annealer for HUBO problems will not be available to the public anytime soon, we now analyze the possible ways to enhance the performance of the D-Wave 2000Q quantum annealer on this problem. After compromising on the maximum two-qubit interactions in the hardware, the subsequent strategy will be to reduce the number of ancilla qubits. With fewer ancilla qubits, the size and accuracy of a solvable network can be improved. As proposed in Ref. [35], the multi-to-two mapping is a general method, but for three-to-two mapping, for example, a more efficient mapping can be constructed with only one ancilla qubit. Suppose there is a sub-Hamiltonian of three-qubit interactions

(10) $${\hat{H}}_{3}=J_{3}{\hat{\sigma }}_{1}^{z}{\hat{\sigma }}_{2}^{z}{\hat{\sigma }}_{3}^{z}.$$

**Figure 8:**
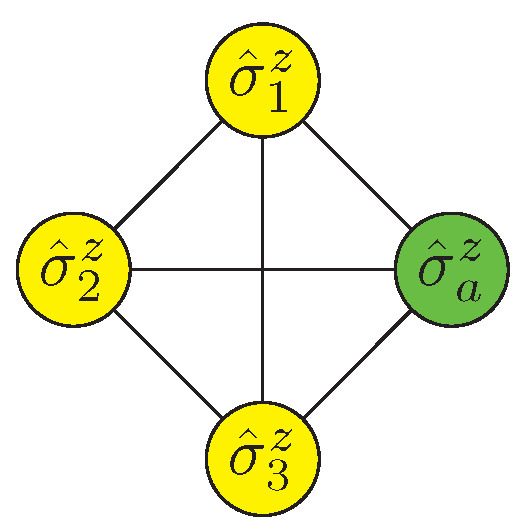
An efficient encoding of three qubits, making use of only one ancilla qubit. The multi-to-two interaction Hamiltonian mapping is a general method, but for three-to-two mapping, a more efficient mapping can be constructed via a subgraph with full connectivity of three logical qubits and one ancilla.

A subgraph with full connectivity of three logical qubits and one ancilla qubit is shown in Figure 8, where the equivalent Hamiltonian is given as

(11) $${\tilde{H}}_{3}=J\sum_{i=2}^{3}\sum_{j=1}^{i-1}{\hat{\sigma }}_{i}^{z}{\hat{\sigma }}_{j}^{z}+h\sum_{i=1}^{3}{\hat{\sigma }}_{i}^{z}+J^{a}\sum_{i=1}^{3}{\hat{\sigma }}_{i}^{z}{\hat{\sigma }}_{a}^{z}+h^{a}{\hat{\sigma }}_{a}^{z}.$$

In contrast to the previous protocol, $J^{a}=2J>h$ and $h^{a}=2h=2J_{3}$. In addition, for sampling out the thermal fluctuation, we take $J^{a}\geq J_{3}$ to prevent the protocol from failing for the same reason. The ancilla qubits can be reduced to about 7000 with this method. Meanwhile, the partition method in *qbsolv* may cause the system to get stuck in local minima that require a better algorithm in the main loop.

## 6. Conclusions

We have implemented the algorithm proposed in Ref. [9] in a D-Wave quantum annealer to solve the equilibrium state of a complex financial network that predicts financial crashes. Although the size of the studied financial network is limited, this proof of principle is in agreement with the results of an exhaustive search. This may be improved with the design of a customized “financial quantum annealer”, a quantum processor with suitable connectivity for the efficient embedding of this kind of problem. Such coherent quantum annealers can be built with current technology [38,39,40], providing convenient multi-qubit couplings.

## Figures and Tables

**Table 1:** Low-energy spectrum (first 8 eigenstates) of the two-qubit Hamiltonian, Equation (7) with k=3, as a result of mapping the term ${\hat{\sigma }}_{1}{\hat{\sigma }}_{2}{\hat{\sigma }}_{3}$ according to Ref. [35]. Values of the parameters $J_{3}$ = 1 u, $J=J_{a}=20\;u$, $q_{0}=10\;u$, h=−10u, $q_{1}=q_{3}=9\;u$, q2=11u, $h_{1}=29\;u$, $h_{2}=-9\;u$, $h_{3}=-51\;u$.

${\hat{\sigma }}_{1}$	${\hat{\sigma }}_{2}$	${\hat{\sigma }}_{3}$	${\hat{\sigma }}_{1}^{a}$	${\hat{\sigma }}_{2}^{a}$	${\hat{\sigma }}_{3}^{a}$	Energy (u)
1	1	−1	−1	−1	1	−121
1	−1	1	−1	−1	1	−121
−1	1	1	−1	−1	1	−121
−1	−1	−1	1	1	1	−121
1	1	1	−1	−1	−1	−119
1	−1	−1	−1	1	1	−119
−1	1	−1	−1	1	1	−119
−1	−1	1	−1	1	1	−119

## Data Availability

The available data that support the findings of this study are available at https://github.com/yongchengding/DWaveFinancialCrashPrediction (accessed on 31 October 2022).
